# Genome-Wide Identification and Expression Analysis of the *YABBY* Gene Family in Bitter Gourd

**DOI:** 10.3390/biology15211938

**Published:** 2026-10-25

**Authors:** Cheng Qiu, Ziqing Guo, Qin Zhou, Qianwen Ge, Xifeng Chen

**Affiliations:** 1 College of Education, Zhejiang Normal University, Jinhua 321004, China; qiuchenggggg@163.com; 2 Jinhua Academy of Agricultural Sciences, Jinhua 321004, China; guoziqing26@163.com (Z.G.); m15888991017@163.com (Q.Z.); 3 College of Life Sciences, Zhejiang Normal University, Jinhua 321004, China

**Keywords:** *Momordica charantia*, *YABBY* gene, genome-wide identification, expression pattern

## Abstract

YABBYs are transcription factors involved in fruit development, leaf polarity establishment, and adaptation to stresses in plants. Bitter gourd (*Momordica charantia* L.) is an important cucurbit crop, but *YABBY* genes have not been reported in this species. In this study, the *YABBY* gene family of bitter gourd was digitally identified using its T2T genome, and the sequence characteristics, phylogenetic relationships, and expression profiles of each gene member were analyzed through bioinformatic and experimental approaches. A total of eight *YABBY* homologous genes, designated *McYABBY1* (*Momordica charantia YABBY1*) to *McYABBY8*, were identified and clustered into five clades in the phylogenetic tree, each showing a clear orthologous relationship to a corresponding *Arabidopsis* gene. RNA-seq data and RT-qPCR revealed distinct expression patterns among *McYABBY* genes across various organs and fruit developmental stages. Notably, *McYABBY3* and *McYABBY8* are actively and constitutively expressed during fruit ripening. Additionally, RT-qPCR showed that members of the *McYABBY* gene family exhibit differential expression in response to abiotic stress, with six up-regulated and two down-regulated under salt stress, and five up-regulated, two down-regulated, and one relatively unchanged under PEG-induced osmotic stress. These findings provide a basis for future functional studies and breeding applications of *McYABBY* genes in bitter gourd.

## Simple Summary

Bitter gourd (*Momordica charantia* L.) is an economically important fruit-type vegetable in the Cucurbitaceae family, yet the molecular mechanisms underlying its fruit development remain largely unexplored. The YABBY transcription factors, which constitute a multi-member gene family, are well-established regulators of fruit development in various plant species. Here, we performed the first genome-wide identification of the *YABBY* gene family in bitter gourd, designating these genes as *McYABBY*. This identification was achieved by screening the recently released high-quality genome for the YABBY-specific conserved domain. We further conducted systematic analyses of the sequence characteristics, phylogenetic relationships, and expression profiles of each *McYABBY* gene member through a combination of bioinformatic and experimental approaches. Our findings reveal both conserved features and functional divergence among the *McYABBY* members, and provide initial insights into their potential roles in fruit development and stress responses. Collectively, this work establishes a solid foundation for future functional studies and molecular breeding efforts targeting the *McYABBY* genes in bitter gourd.

## 1. Introduction

YABBYs are well-known plant-specific transcription factors. Structurally, YABBY proteins possess two conserved domains: an N-terminal C_2_C_2_ zinc finger domain and a C-terminal helix–loop–helix domain, commonly referred to as the YABBY domain [1]. Functionally, YABBYs are essential for maintaining leaf adaxial–abaxial polarity and play key roles in regulating vegetative and reproductive growth, hormone responses, stress resistance, and secondary metabolite biosynthesis [2]. Notably, accumulating evidence supports an indispensable role for YABBYs in fruit development.

The first *YABBY* gene, *CRABS CLAW* (*CRC*), was cloned from the model plant *Arabidopsis thaliana* [3] and positively regulates carpel polarity and nectary development [4]. Subsequently, five additional *YABBY* genes *FIL* (*AtYABBY1)*, *YAB2* (*AtYABBY2*), *YAB3* (*AtYABBY3*), *INO* (*AtYABBY4*) and *YAB5* (*AtYABBY5*) were identified in *Arabidopsis*, each playing distinct roles at different developmental stages [1]. To date, genome-wide analyses have shown that YABBYs are widely distributed across plant species. For example, eight *YABBY* genes have been identified in rice (*Oryza sativa*) [5], nine in tomato (*Solanum lycopersicum*) [6], nine in pineapple (*Ananas comosus*) [7], 21 in wheat (*Triticum aestivum*) [8], six in pomegranate (*Punica granatum*) [9], and nine in watermelon (*Citrullus lanatus*) [10]. In tomato, *YABBY* genes have been shown to be important regulators of fruit development [11]. Overexpression of *SlCRCa* in tomato resulted in reduced fruit size, as *SlCRCa* negatively regulates fruit size by influencing pericarp cell division and cell expansion [12]. *SlYABBY2a* was specifically highly expressed in the tomato fruit septum. In the *slyabby2a* mutant septum developmental defects were observed, including inward depression of the pericarp and delayed septum ripening [13]. Overexpression of *SlYABBY2b* led to reduced locule number and decreased fruit weight in tomato [14]. In cucurbit crops, the regulatory roles of *YABBY* genes in fruit development have been progressively revealed. In cucumber (*Cucumis sativus*), *CsCRC* directly targets and upregulates the auxin-responsive gene *CsARP1*. Overexpression of the *CsCRC^G^* allele resulted in a 9% increase in fruit length [15]. In melon (*Cucumis melo*), overexpression of *CmYAB2* significantly increased both fruit length and diameter by influencing genes associated with cell division and expansion [16].

Studies have shown that the expression of *YABBY* genes is affected by various environmental factors, including drought, high salinity, and low temperature. In rice, overexpression of *OsYABBY6* upregulated the expression of cold-responsive genes such as *OsDREB1A*/*B*/*C*, thereby enhancing cold tolerance. Compared with wild-type plants, *Osyabby6* mutants exhibited reduced water loss, lower reactive oxygen species (ROS) levels, and greater accumulation of proline and soluble sugars, consistent with enhanced drought tolerance [17]. In pineapple, *AcYABBY4* negatively regulates plant salt tolerance. In transgenic *Arabidopsis* plants heterologously overexpressing *AcYABBY4*, primary root growth was significantly inhibited under NaCl treatment [7]. In soybean (*Glycine max*), *GmYABBY3*, *GmYABBY10*, and *GmYABBY16* negatively regulate plant tolerance to drought and salt stress [18]. In wheat, the expression of all *YABBY* family members was induced within a short period under salt stress [8]. In maize (*Zea mays*), *YABBY* genes responded to nutrient stress conditions, including low-phosphorus and high-nitrogen conditions [19], and *ZmYABBY8* was significantly induced under drought stress [20].

Bitter gourd (*Momordica charantia* L.) is a tropical and subtropical vine belonging to the family Cucurbitaceae, genus *Momordica* [21]. The fruit of bitter gourd is not only an important vegetable but also possesses various health-promoting properties, including appetite enhancement, digestion promotion, cardiovascular benefits, and anti-inflammatory effects. Thus, bitter gourd is a multi-functional medicinal crop with considerable nutritional and health value, playing important roles in both agriculture and medicine [22]. Fruit yield and quality are important economic traits in bitter gourd. Additionally, the productivity of cucurbit crops is severely constrained by drought and salt stress [23]. So far, the *YABBY* genes have not been reported in bitter gourd. Recently, the high-quality genome of bitter gourd at the telomere-to-telomere (T2T) level was just published [24], which provides an opportunity for systematic investigation of the *YABBY* gene family in this species. In this study, we identified the *YABBY* gene family in bitter gourd through multiple bioinformatics approaches, including gene structure characterization, three-dimensional protein modeling, conserved motif identification, chromosomal localization, multiple sequence alignment, and collinearity analysis. In addition, the expression patterns of *YABBY* genes in different organs of bitter gourd and under abiotic stresses were examined in our experiments. These findings provide a foundation for future functional studies and molecular breeding of *YABBY* genes in bitter gourd.

## 2. Materials and Methods

### 2.1. Plant Materials and Growth Conditions

The bitter gourd cultivar ‘Biyue’ was bred by the Jinhua Academy of Agricultural Sciences, Zhejiang Province, China. After germination, the bitter gourd seeds were sown in the field in Jinhua City in March under conventional management practices. Roots, stems, leaves, and flowers were collected at the flowering stage. Fruits were sampled at 4, 7, 12, 15, and 18 days after pollination. All collected organs were immediately frozen in liquid nitrogen and subsequently stored at −80 °C.

For stress treatments, the bitter gourd seeds were grown in pots with soil in a growth chamber at 25 °C under a light intensity of 400 μmol photons m^−2^ s^−1^ (8 h dark/16 h light). At the four-leaf stage, the pots were then immersed in 20% polyethylene glycol 6000 (PEG-6000) solution for osmotic-stress treatment or in 205 mM NaCl solution for salt stress treatment, respectively. Whole seedlings were sampled at 0, 6, 12, and 24 h after treatment. Three biological replicates were collected at each time point, with six randomly selected seedlings pooled for each replicate. Samples were rapidly frozen in liquid nitrogen and stored at −80 °C.

### 2.2. Identification of the YABBY Gene Family in Bitter Gourd

The genome assembly (Y52. genome. v1. fasta), predicted protein sequences (Y52. genome. v1. protein fasta), and GFF3 annotation file (Y52. genome. v1. gff3) of bitter gourd cultivar Y52 were downloaded from the Bittergourd DB (http://bittergourddb.omicsplant.cn/, accessed on 13 April 2026). The protein sequences of *Arabidopsis* YABBY were used as queries to search the predicted proteome of bitter gourd by BLAST v2.17.0 with an E-value ≤ 1 × 10^−5^ set for homologous sequence filtering. To improve sensitivity, the Hidden Markov Model (HMM) configuration file for the YABBY domain (PF04690) was downloaded from the Pfam database (http://pfam.xfam.org, accessed on 13 April 2026) and the same proteome was searched using HMMER V3.2.2 [25] (http://hmmer.org/, accessed on 13 April 2026) with an E-value ≤ 1 × 10^−5^. The BLAST and HMMER hits were then merged and cross-validated. All remaining non-redundant candidate proteins were subsequently examined for the presence of both the C_2_C_2_ zinc-finger domain and the YABBY domain using SMART (http://smart.embl-heidelberg.de/, accessed on 13 April 2026) [26]. Subcellular localization of the identified proteins was predicted using Cell-PLoc (http://www.csbio.sjtu.edu.cn/bioinf/Cell-PLoc-2/, accessed on 13 April 2026) [27]. The GFF3 file of the bitter gourd genome was used to visualize gene structures and chromosomal locations using TBtools v2.420 [28]. Conserved motifs of the YABBY proteins were identified using MEME (https://meme-suite.org/meme/, accessed on 13 April 2026) [29]. The three-dimensional structures of the proteins were predicted using Phyre2.2 (https://www.sbg.bio.ic.ac.uk/phyre2/, accessed on 13 April 2026) [30].

### 2.3. Phylogenetic and Syntenic Analysis

The sequences of YABBY family proteins from several plant species, including six from *Arabidopsis*, eight from rice, six from strawberry (*Fragaria vesca*), six from pomegranate (*Punica granatum*), nine from tomato, and nine from watermelon, were downloaded from PlantTFDB (https://planttfdb.gao-lab.org/, accessed on 13 April 2026). These YABBY proteins were aligned with bitter gourd YABBY proteins using MEGA12 [31] and a phylogenetic tree was constructed using the neighbor-joining (NJ) method with the bootstrap value set to 1000. For synteny analysis, the *Arabidopsis* genome data were downloaded from TAIR (https://www.arabidopsis.org/, accessed on 13 April 2026) and the bitter gourd genome data were obtained from the Bittergourd DB (http://bittergourddb.omicsplant.cn/, accessed on 13 April 2026). Intra-species collinearity analysis among bitter gourd chromosomes and inter-species collinearity analysis of *YABBY* genes between bitter gourd and *Arabidopsis* were performed using MCScanX v1 [32], and the resulting collinear gene pairs were visualized using TBtools v2.420 [28].

### 2.4. Gene Expression Analysis Based on RNA-Seq Data

RNA sequencing (RNA-Seq) data from the leaves, flowers, small fruits, and big fruits of bitter gourd were downloaded from the Bittergourd DB (http://bittergourddb.omicsplant.cn/, accessed on 13 April 2026). The Transcripts Per Million (TPM) values of bitter gourd *YABBY* genes were extracted and log2 transformed as log2 (TPM+1). A heatmap of gene expression was generated using TBtools v2.420.

### 2.5. Gene Expression Assay by RT-qPCR

Total RNA was extracted using the EASYspin Plus Plant RNA Rapid Extraction Kit (Aidlab, Beijing, China). First-strand cDNA was synthesized using the PrimeScript RT Reagent Kit (TaKaRa, Kusatsu, Japan) according to the manufacturer’s instructions. qPCR was performed using the TB Green Premix Ex Taq™ II (Tli RNaseH Plus) Kit (TaKaRa, Kusatsu, Japan). The reaction mixture had a final volume of 10 µL and contained 5 µL of 2× SYBR Premix Ex Taq™ II, 3.3 µL of ddH_2_O, 0.5 µL of gene-specific primers (Table 1), 0.2 µL of ROX Reference Dye II, and 1 µL of cDNA template. qPCR was run on an ABI 7500 Fast Real-Time PCR System (ABI, Vernon, CA, USA) using the following program: 95 °C for 5 min, followed by 40 cycles of 95 °C for 15 s and 60 °C for 30 s. The bitter gourd *Actin* gene was used as the internal reference [33]. Each sample was analyzed in three biological replicates. Relative gene expression levels were calculated using the 2^−ΔΔCT^ method [34].

## 3. Results

### 3.1. Identification of the McYABBY Gene Family in Bitter Gourd

**Figure 1:**
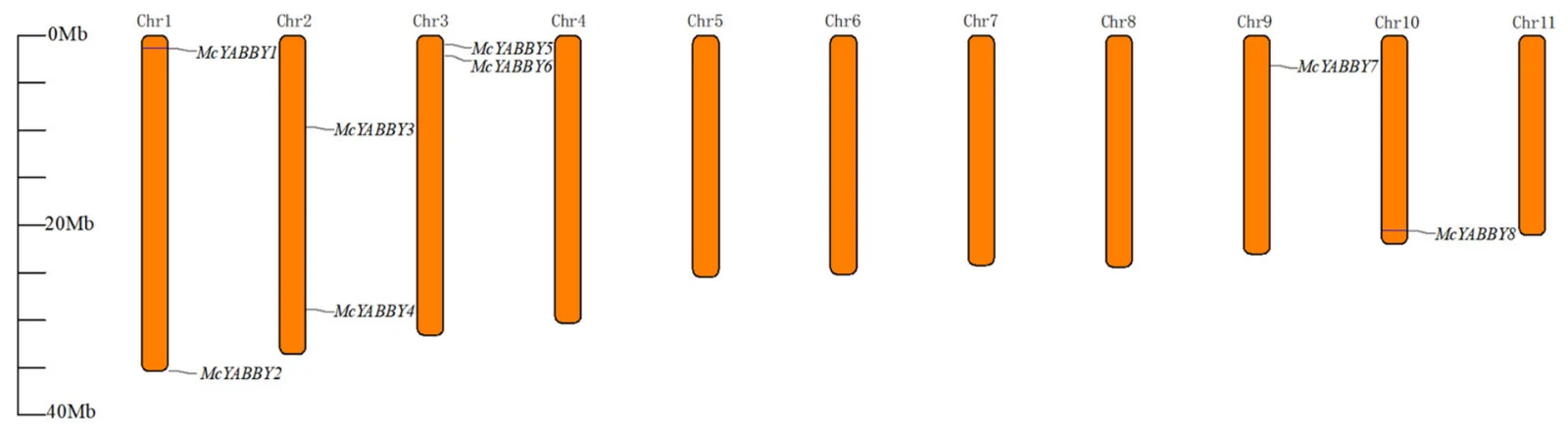
Chromosome distribution of *McYABBY* genes in bitter gourd.

Using *Arabidopsis* YABBY proteins as queries, a total of eight *YABBY* homologous genes were identified in the bitter gourd genome. These genes were distributed on five of the eleven chromosomes (Chr) and were named *McYABBY1* to *McYABBY8* according to their chromosome order (Table 2). Specifically, *McYABBY1* and *McYABBY2* were located on Chr 1; *McYABBY3* and *McYABBY4* were located on Chr 2; *McYABBY5* and *McYABBY6* were located on Chr 3; and *McYABBY7* and *McYABBY8* were located on Chr 9 and Chr 10, respectively (Figure 1). The genomic lengths of the *McYABBY* genes ranged from 1 to 6 kb. The coding sequence (CDS) lengths of the *McYABBY* genes ranged from 510 to 699 bp, encoding proteins of 169 to 232 amino acids. Subcellular localization prediction showed that all of the McYABBY proteins were predicted to localize to the nucleus (Table 2).

### 3.2. Gene Structure and Encoding Protein Architecture of McYABBYs

**Figure 2:**
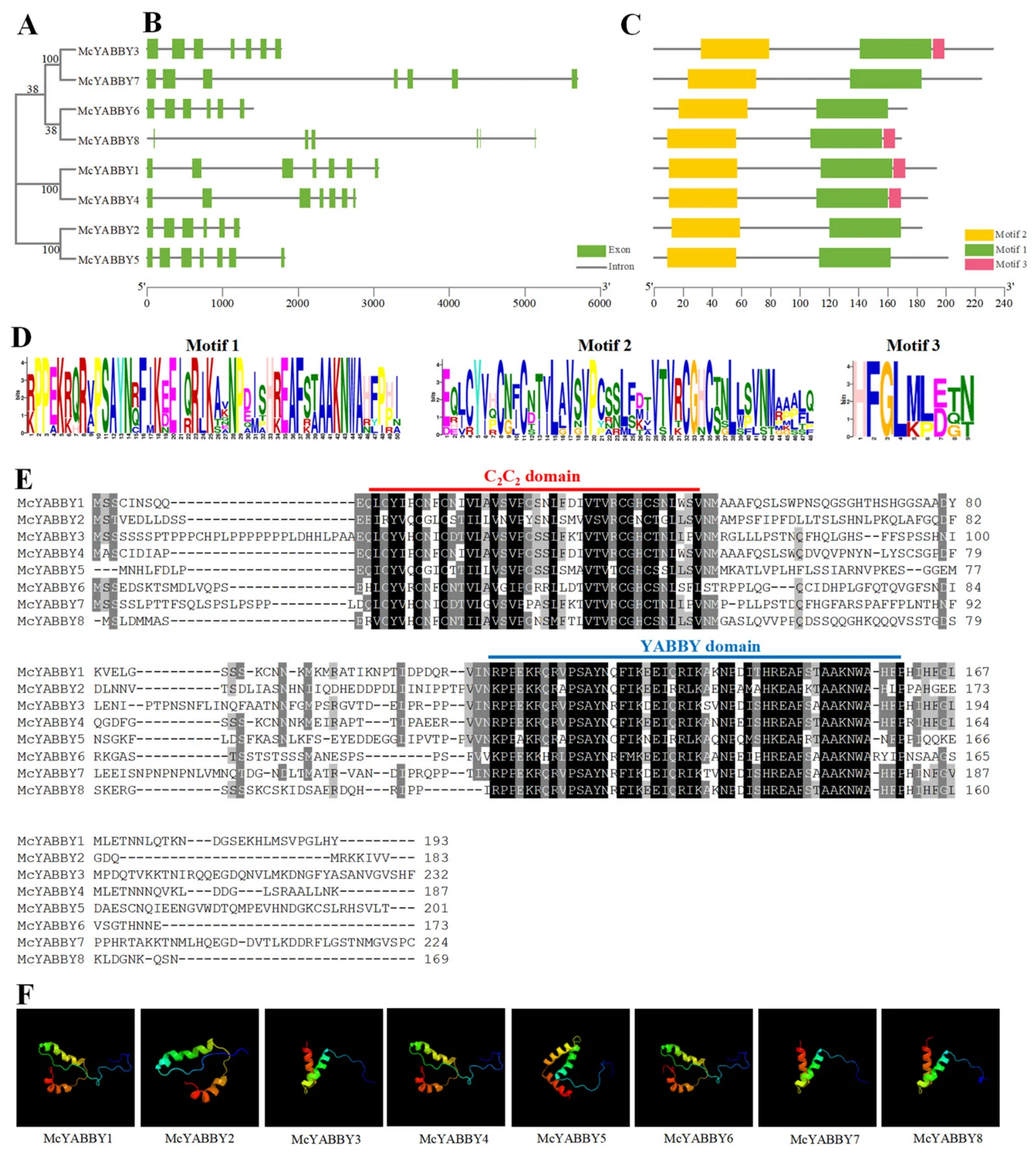
Sequence characters of *McYABBY* genes and their encoding proteins. (**A**) The cluster tree of McYABBYs. (**B**) The gene structures of *McYABBYs*. (**C**) The conserved motif compositions of McYABBYs. (**D**) Seqlogo diagram of the conserved motifs. (**E**) Amino-acid sequence alignment of the C_2_C_2_ zinc finger domain and YABBY domain from McYABBYs. The conserved residues are boxed in black or light gray based on the degree of conservation. (**F**) Three-dimensional structure prediction of McYABBYs by Phyre 2.0.

Based on the similarity of their deduced protein sequences, the eight *McYABBYs* could be classified into four groups consisting of the gene pairs *McYABBY1*/*4*, *McYABBY2*/*5*, *McYABBY3*/*7*, and *McYABBY6*/*8* (Figure 2A). Gene structure analysis showed that all *McYABBY* genes contained multiple introns, with numbers ranging from five to six (Figure 2B). Amino acid sequence analysis showed that all McYABBY proteins contained two conserved motifs (Figure 2C,D): an N-terminal C_2_C_2_ zinc-finger domain in motif 1 and a highly conserved C-terminal YABBY domain in motif 2 (Figure 2E). Additionally, McYABBY1/3/4/8 proteins were predicted to contain an additional unknown motif in their C-terminal regions (Figure 2C,D). The three-dimensional structures of McYABBY proteins were mainly composed of random coils, followed by α-helices and β-turns (Figure 2F).

### 3.3. Syntenic and Phylogenetic Analysis of McYABBYs

**Figure 3:**
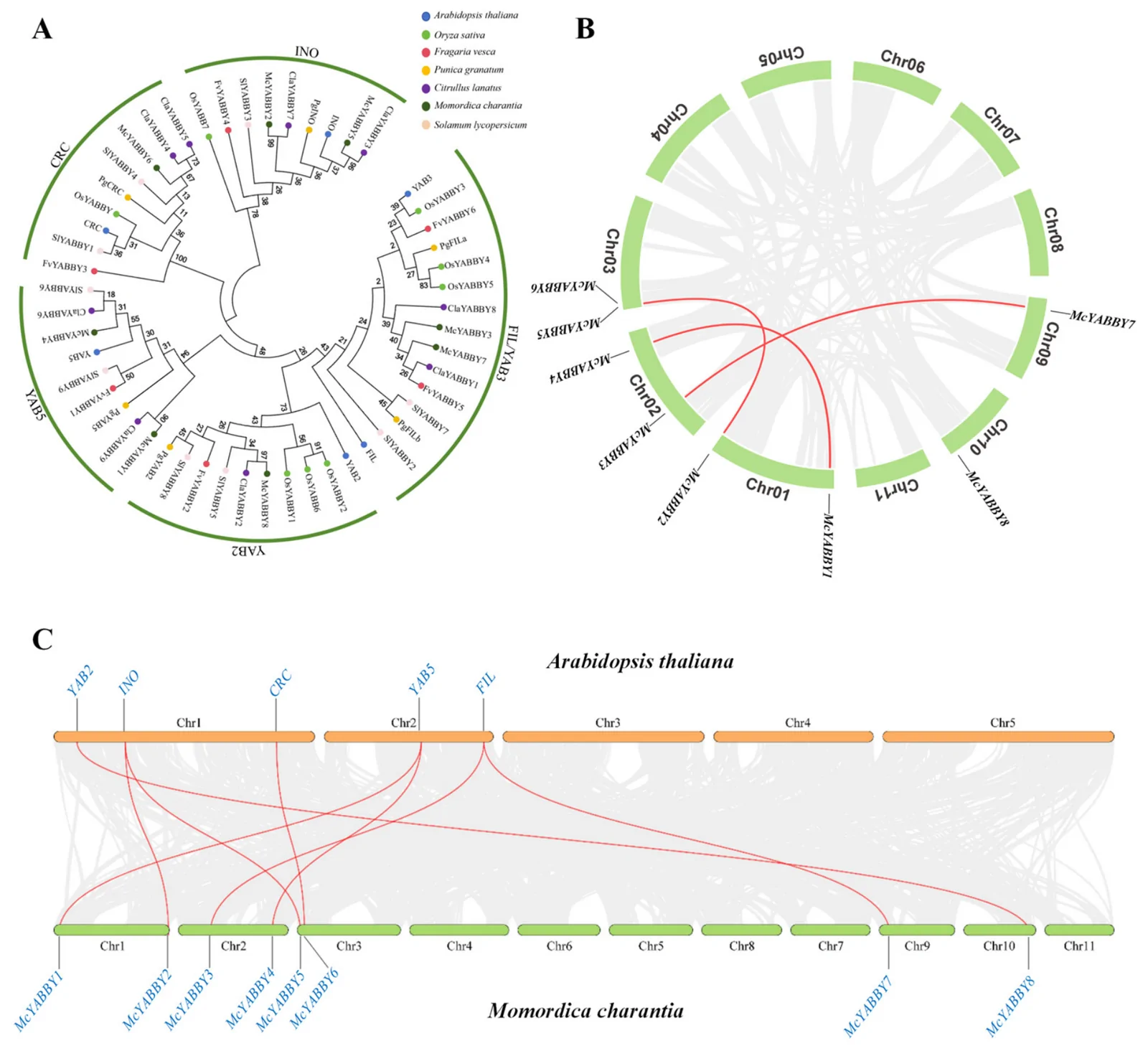
(**A**) Phylogenetic tree of *McYABBY* gene families. (**B**) Intra-species collinearity analysis of *McYABBY* genes across the 11 chromosomes of bitter gourd (Chr01–Chr11). Gray lines in the background indicate all syntenic blocks in the bitter gourd genome, and red lines highlight the segmentally duplicated *McYABBY* gene pairs. (**C**) Collinearity of *YABBY* genes between bitter gourd and *Arabidopsis*. Yellow boxes (Chr1-Chr5) refer to the 5 chromosomes of *Arabidopsis*, and green boxes (Chr1-Chr11) refer to 11 chromosomes of bitter gourd. Gray lines refer to the collinear region in the genomes of bitter gourd and *Arabidopsis*, and red lines highlight the collinear *YABBY* gene pairs.

To elucidate the evolutionary relationships among the *McYABBY* genes, a phylogenetic tree was constructed based on the deduced protein sequences of 52 YABBY homologs from bitter gourd, *Arabidopsis*, rice, strawberry, pomegranate, tomato, and watermelon. The resulting tree showed that these YABBY proteins could be divided into five canonical clades: CRC, FIL/YAB3, YAB2, YAB5, and INO (Figure 3A). All McYABBYs were resolved into the five clades, with no lineage forming an independent branch, a topology consistent with the previously established five-clade classification of the YABBY family in plants. Among them, McYABBY6 and McYABBY8 were assigned to clade CRC and YAB2, respectively. The remaining McYABBYs were distributed into the other three clades, with McYABBY2/5 in clade INO, McYABBY3/7 in clade FIL/YAB3, and McYABBY1/4 in clade YAB5. The phylogenetic results were largely in agreement with the grouping based on the deduced protein sequence similarity of McYABBYs (Figure 2A). In addition, McYABBYs clustered more closely with dicot YABBYs than with monocot ones, particularly with those from other cucurbit crops, which is consistent with their phylogenetic background (Figure 3A).

To further investigate the evolutionary expansion mechanism of the *McYABBY* gene family, an intra-species collinearity analysis among the 11 chromosomes of bitter gourd was conducted (Figure 3B). The results revealed a total of 3 collinear *McYABBY* gene pairs—namely *McYABBY1*/*4* (located on Chr 1 and Chr 2), *McYABBY2*/*5* (on Chr 1 and Chr 3), and *McYABBY3*/*7* (on Chr 2 and Chr 9)—situated within conserved syntenic blocks across different chromosomes. In contrast, no collinear relationships were detected for *McYABBY6* (on Chr 3) and *McYABBY8* (on Chr 10). Moreover, although *McYABBY5* and *McYABBY6* were both located on Chr 3, they were separated by more than 1.15 Mb and showed no tandem duplication relationship. These findings demonstrate that the expansion of the *McYABBY* gene family in bitter gourd arose exclusively from segmental duplication rather than tandem duplication. Notably, the intra-species segmental duplication patterns were highly consistent with the phylogenetic topologies. The three segmentally duplicated paralogous pairs (*McYABBY1*/*4*, *McYABBY2*/*5*, and *McYABBY3*/*7*) shared high sequence homology and clustered tightly together into the same respective clades—YAB5, INO, and FIL/YAB3. Conversely, the non-duplicated members *McYABBY6* and *McYABBY8* were individually resolved into the CRC and YAB2 clades, respectively.

Furthermore, an inter-species syntenic analysis between the bitter gourd and *Arabidopsis* genomes revealed that the eight *McYABBY*s were collinear with five *Arabidopsis YABBYs* (Figure 3C). Among them, *McYABBY6* and *McYABBY8* each had a single ortholog in *Arabidopsis*, corresponding to *CRC* and *YAB2*, respectively. In contrast, the three segmentally duplicated paralogous gene pairs (*McYABBY1/4*, *McYABBY2/5*, and *McYABBY3/7*) each corresponded to a single *Arabidopsis* ortholog, namely *YAB5*, *INO*, and *FIL*, respectively. Notably, the intra- and inter-species synteny assignments were fully consistent with the phylogenetic results (Figure 3A–C), and this reciprocal correspondence further supports the reliability of both the phylogenetic and synteny analyses.

### 3.4. Expression Pattern of McYABBYs in Different Organs

**Figure 4:**
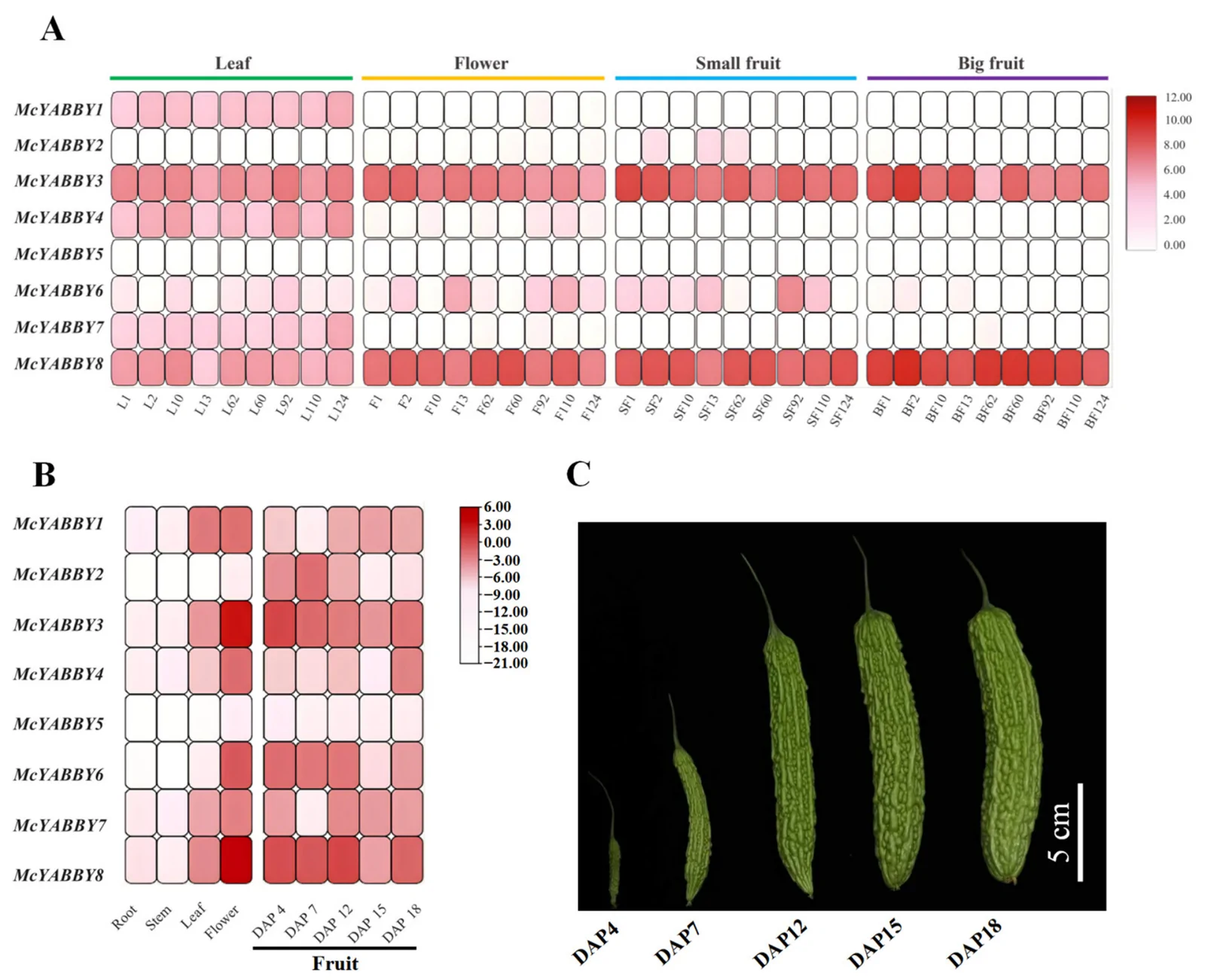
Expression pattern of the *McYABBY* gene family in bitter gourd’s organs. (**A**) Nine bitter gourd cultivars’ *YABBY* gene expression level in different organs and fruit developmental stages was analyzed by RNA-seq from the database (http://bittergourddb.omicsplant.cn/). (**B**) Gene expression patterns in root, stem, leaf, flower sampled during the flowering period, and different development stages of fruit by RT-qPCR, using log2-transformed expression data to present the results. (**C**) The fruits of bitter gourd at different development stages. DAP refers to days after pollination.

To investigate the expression patterns of the eight *McYABBY* genes, we first analyzed their organ-specific expression using RNA-seq data from the database. The dataset included expression profiles from bitter gourd leaves, flowers, and fruits at two developmental stages (Table S1). The results showed that *McYABBY3* and *McYABBY8* were highly expressed across different organs and fruit developmental stages, whereas *McYABBY2* and *McYABBY5* exhibited extremely low transcript levels. In addition, *McYABBY1*, *McYABBY4*, and *McYABBY7* showed moderate expression mainly in leaves, while *McYABBY6* was expressed in flowers and young fruits (Figure 4A). To validate the reliability of the RNA-seq data, RT-qPCR was performed on the eight *McYABBY* genes. The RT-qPCR results independently supported the overall expression trends observed in the RNA-seq dataset (Figure 4B,C). Together, these results revealed marked expression divergence among *McYABBY* family members during the development of different organs in bitter gourd.

### 3.5. Expression Patterns of McYABBYs Under Salt- and PEG-Induced Osmotic Stress

**Figure 5:**
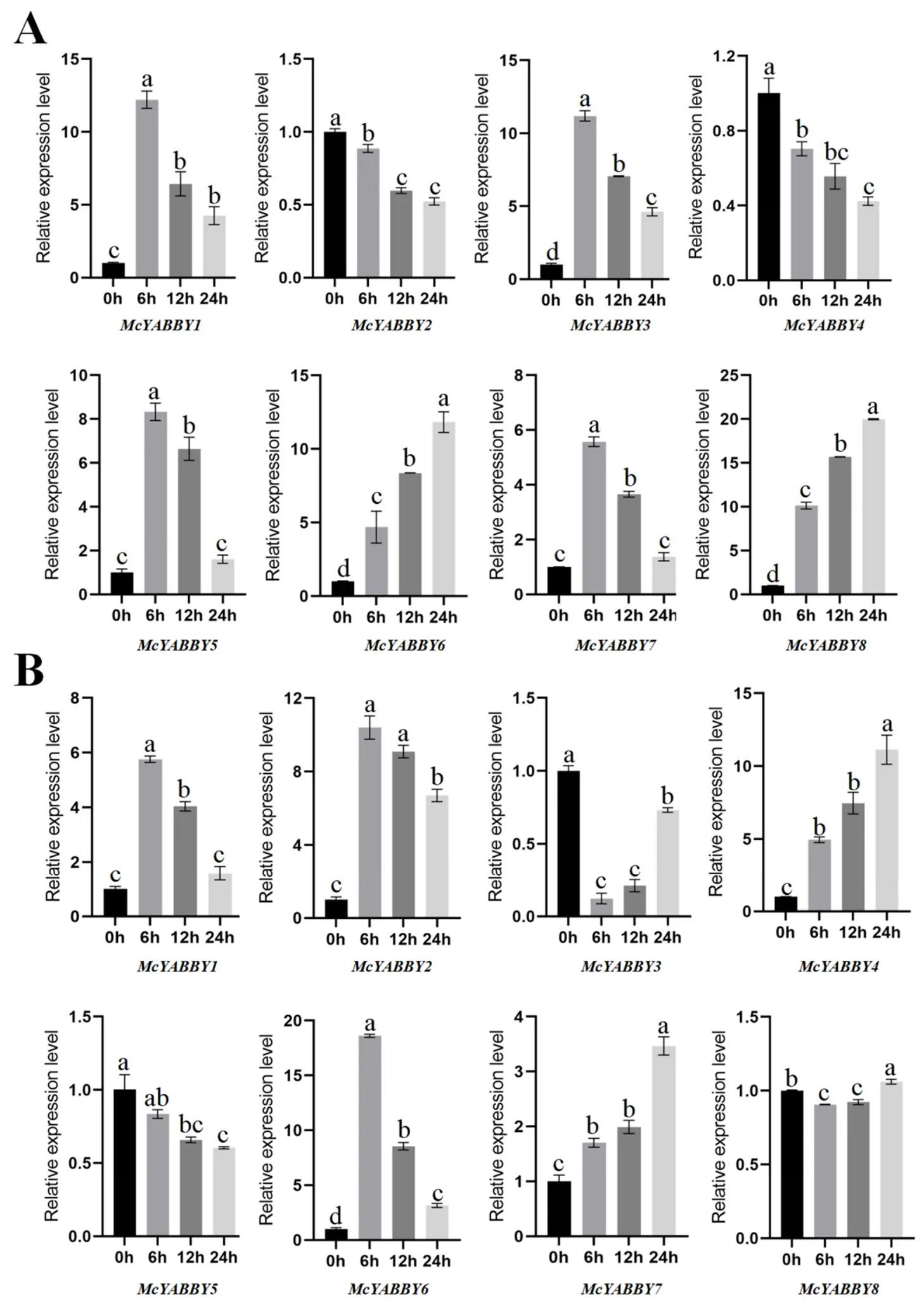
Expression assay of *McYABBYs* under salt and PEG-induced osmotic stress by RT-qPCR. The four-leaf stage bitter gourd seedlings were subjected to salt (**A**) and PEG-induced osmotic (**B**) stress at 0, 6, 12 and 24 h. Error bars denote standard deviations from three biological replicates. Different letters above bars represent statistically significant differences between groups (*p* < 0.05) as determined by one-way ANOVA followed by Tukey’s HSD post hoc test.

To further investigate the expression patterns of *McYABBYs* under different abiotic stresses, RT-qPCR was used to examine their relative expression levels after salt- and PEG-induced osmotic treatments for 0, 6, 12, and 24 h. The results showed that members of the *McYABBY* gene family exhibited differential expression in response to abiotic stresses. Under salt stress, the expression levels of *McYABBY1*, *McYABBY3*, *McYABBY5*, *McYABBY6*, *McYABBY7*, and *McYABBY8* were induced. Among them, *McYABBY1*, *McYABBY3*, *McYABBY5*, and *McYABBY7* showed more rapid responses to salt stress, reaching peak expression at 6 h and then gradually decreasing. In contrast, *McYABBY6* and *McYABBY8* exhibited a gradual increase in expression over time. Notably, the expression level of *McYABBY8* at 24 h was more than 20-fold higher than that at 0 h, consistent with a strong late-stage cumulative response. Conversely, *McYABBY2* and *McYABBY4* were markedly repressed under salt stress (Figure 5A).

Under PEG-induced osmotic stress, the response patterns of *McYABBY* genes were more diverse. *McYABBY1*, *McYABBY2*, and *McYABBY6* displayed typical early stress-responsive expression characteristics, with their transcript levels increasing sharply after 6 h of PEG-induced osmotic treatment and then rapidly declining. *McYABBY4* and *McYABBY7* showed a gradual upregulation trend as the duration of PEG-induced osmotic treatment increased. By contrast, the expression levels of *McYABBY3* and *McYABBY5* were significantly suppressed under PEG-induced osmotic stress. Interestingly, *McYABBY8* maintained a relatively stable expression level throughout the PEG-induced osmotic treatment period (Figure 5B).

## 4. Discussion

The *YABBY* gene family is a plant-specific transcription factor family that plays important regulatory roles in the establishment of leaf polarity, lateral organ development, and the formation of floral organs and ovules. In this study, we identified eight *YABBY* genes in bitter gourd, a number comparable to that in rice. The eight *McYABBY* genes were unevenly distributed on chromosomes 1, 2, 3, 9, and 10. Protein structure analysis revealed that all eight McYABBY proteins contained the typical C_2_C_2_ zinc-finger domain and YABBY domain. Among them, *McYABBY1*, *McYABBY3*, *McYABBY4*, and *McYABBY8* all possessed motif 3. Gene structure analysis revealed that all eight *McYABBY* genes contained multiple intron–exon structures, and members within the same subgroup exhibited relatively similar structural features. These findings are consistent with a closer phylogenetic relationship among these four genes during evolution, particularly between *McYABBY1* and *McYABBY4*. Overall, these results are consistent with the *McYABBY* family having maintained a high degree of evolutionary conservation while also undergoing some structural differentiation during evolution.

Phylogenetic analysis showed that bitter gourd YABBY proteins could be clearly classified into five canonical clades: CRC, FIL/YAB3, YAB2, YAB5, and INO. No bitter gourd-specific novel clade was observed. However, the number of members in different clades was uneven. Notably, McYABBYs preferentially clustered with ClaYABBYs into closely related subclades and, overall, were more closely grouped with YABBY proteins from dicotyledonous species such as tomato. This clustering pattern is consistent with the taxonomic relationships among these species and with the higher sequence similarity of *YABBY* genes among cucurbit crops. Synteny analysis revealed both one-to-one and one-to-many orthologous relationships between *Arabidopsis* and bitter gourd. Furthermore, gene duplication events, primarily encompassing segmental and tandem duplications, serve as major driving forces for gene family expansion and functional diversification in plants. Our intra-species collinearity analysis demonstrated that the expansion of the *McYABBY* family in bitter gourd was driven exclusively by segmental duplication rather than tandem duplication, yielding three paralogous gene pairs (*McYABBY1*/*4*, *McYABBY2*/*5*, and *McYABBY3*/*7*) across different chromosomes (Figure 3B). Integrating the collinearity data with the phylogenetic tree provides clear evolutionary insights: each of these three segmentally duplicated pairs exhibited high sequence homology and clustered tightly onto the same branch within clades YAB5, INO, and FIL/YAB3, respectively. Moreover, each duplicated pair in bitter gourd shared synteny with a single ancestral ortholog in *Arabidopsis* (*YAB5*, *INO*, and *FIL*, respectively), whereas *McYABBY6* (clade CRC) and *McYABBY8* (clade YAB2) did not undergo segmental duplication and maintained strict one-to-one orthology with *Arabidopsis CRC* and *YAB2*. This strong concordance between intra-species segmental duplication and phylogenetic clustering reveals that these three paralogous pairs originated from chromosomal segmental duplication events and retained high structural conservation during evolution.

Based on the RNA-seq and RT-qPCR results, the *McYABBY* gene family exhibited clear expression differentiation among different vegetative and reproductive organs of bitter gourd. The broad expression of *McYABBY3* and *McYABBY8* is consistent with their potential involvement in basic organ morphogenesis, in line with the core function of *YABBY* genes in establishing abaxial polarity in lateral organs [35]. In contrast, the leaf-specific expression of *McYABBY1*, *McYABBY4*, and *McYABBY7* is consistent with their potential involvement in leaf development. Similar organ-specific expression patterns have also been observed in other species. For example, in watermelon, *YABBY* genes are divided into several subfamilies, and some members show preferential expression in floral organs or vegetative tissues [10]. In addition, the expression of *McYABBY6* in flowers and young fruits is consistent with the general role of YABBY family members in reproductive organ development [8]. By contrast, *McYABBY2* and *McYABBY5* maintained relatively low expression levels overall. Both genes showed collinearity with *Arabidopsis INO*, which is predominantly expressed on the abaxial side of the outer integument of the ovule and plays a critical role in the asymmetric growth of the outer integument and ovule development [36]. Therefore, it was speculated that the low expression levels of *McYABBY*2 and *McYABBY*5 at the organ level may not necessarily reflect a loss of biological function. Instead, these genes may be expressed in ovules or integuments, or at specific stages of seed development.

Notably, *McYABBY3* and *McYABBY8* were not only highly expressed in non-fruit organs but also maintained extremely high expression levels throughout fruit development in bitter gourd. This finding is highly consistent with studies in cucurbit crops. For instance, in cucumber, *CsYABBY* genes have been shown to coordinate parthenocarpy and fruit development through spatiotemporal expression patterns, with certain members exhibiting constitutively high expression [37]. Studies on the watermelon YABBY family have also shown that *ClaYABBY2* is continuously highly expressed throughout fruit development and is considered a core regulator of watermelon fruit development [10]. In addition, cross-species comparative studies on the functional conservation of the YABBY family have reported sustained high expression of specific members during fruit development in pineapple (*AcYABBY3*) [7], tomato (*SlYABBY5*) [38], and *Averrhoa carambola* (*AcYABBY3* and *AcYABBY4*) [39], further supporting the conserved role of *YABBY* members in fruit development. In contrast, *McYABBY6* was expressed mainly in flowers and young fruits, reflecting spatiotemporal expression differentiation within the YABBY family during fruit development. The distinction between “broad and sustained expression” and “stage-specific transient accumulation” is consistent with the general regulatory pattern by which *YABBY* genes coordinate fruit development through dynamic spatiotemporal expression. Therefore, *McYABBY3* and *McYABBY8* may be candidate genes associated with fruit development. In addition to their roles in organ development, increasing evidence indicates that the expression of development-related transcription factors can also be regulated by external environmental signals, thereby coordinating plant growth and stress adaptation. In this study, all eight *McYABBY* genes exhibited differential expression in response to salt- or PEG-induced osmotic stress. For example, *McYABBY2* and *McYABBY4* were repressed under salt treatment but induced under PEG-induced osmotic stress, whereas *McYABBY3* and *McYABBY5* showed opposite patterns, being induced by salt stress but suppressed by PEG-induced osmotic stress. These expression patterns support further investigation into the roles of *McYABBY* family members in different stages or types of stress signaling. Notably, *McYABBY8* was broadly and highly expressed under normal conditions and showed strong late-stage induction under salt stress, while remaining relatively stable during PEG-induced osmotic treatment. This response pattern may not simply result from the osmotic effects shared by both salt and PEG-induced osmotic stress, but may instead be associated with salt-specific ion toxicity, reactive oxygen species homeostasis, or related hormonal signaling pathways.

However, two methodological caveats should be noted. First, the PEG-6000 treatment was applied via root drenching in soil, which differs from pure hydroponic systems or natural water deficit; high-viscosity PEG in a solid substrate can impede aeration and alter hydraulic conductivity. Therefore, the expression data should be interpreted strictly as an acute transcriptional response to osmotic shock at the molecular level, rather than a definitive reflection of whole-plant physiological drought responses. Future physiological phenotyping of prioritized candidate genes, particularly McYABBY8, will be conducted in hydroponic or controlled progressive soil-drying systems alongside precise water potential monitoring. Second, cellular ion status (e.g., Na^+^ and K^+^ accumulation or Na^+^/K^+^ ratios) was not monitored. Although transcriptional induction confirms that early stress signaling was triggered, the lack of ion-homeostasis profiling limits our conclusions to the molecular transcript level. Therefore, *McYABBY8* represents a priority candidate for future studies on salt-stress response mechanisms in bitter gourd. In our future work, functionally dissecting the prioritized candidate gene *McYABBY8* through transgenic approaches will combine detailed molecular assays with comprehensive analyses of plant water relations and Na^+^/K^+^ ion balance to clarify its role in bitter gourd salt-stress responses.

Overall, this study revealed that the *YABBY* gene family in bitter gourd is highly conserved in terms of gene structure and evolutionary relationships, but exhibits obvious divergence in expression patterns. These findings point to potential conserved and specific roles of *McYABBY* genes during the development of different organs in bitter gourd. The results not only enrich current knowledge of the YABBY family in cucurbit crops, but also provide valuable candidate gene resources for further elucidating the molecular mechanisms underlying organ development and for improving related agronomic traits in bitter gourd.

## 5. Conclusions

In this study, the YABBY transcription factor family was systematically characterized for the first time in bitter gourd through genome-wide identification and expression analysis. A total of eight *McYABBY* genes were identified, and their structural features, phylogenetic relationships, and expression divergence across organs, fruit developmental stages, and abiotic stress conditions were comprehensively analyzed. Our findings highlight *McYABBY3* and *McYABBY8* as candidate genes for functional studies on fruit development regulation, given their constitutively high expression throughout the entire fruit development process, a pattern that has been observed for functionally important YABBY members in other cucurbit crops. Furthermore, members of the *McYABBY* gene family exhibit differential expression in response to abiotic factors. These results not only enrich our understanding of the evolutionary conservation and functional diversification of the *YABBY* gene family in cucurbit crops but also provide valuable candidate genes for improving fruit traits and stress responses in bitter gourd.

## Figures and Tables

**Table 1:** Sequences of primer pair used for RT-qPCR.

Gene	Forward Primer (5’→3’)	Reverse Primer (5’→3’)	Amplicon Length (bp)
*McActin*	CCCTCCCTCATGCAATTCTC	TCGGCAGTAGTGGTAAACATGTAAC	124
*McYABBY1*	ATCAATAGTCAGCAGGAGCA	GACGGTCACGATGTCAAAC	105
*McYABBY2*	ATGGTGGTGAGCGTTAGATG	AGGTCGGATGTGACGTTGT	164
*McYABBY3*	CTCCAACTCCTCCACCCTG	AAGGAACACTCACCGCAAG	135
*McYABBY4*	CCTCCTCCAAATGTAACAACA	AGCTTCCCTATGGCTAATCTC	185
*McYABBY5*	CCACTGCTCAAGCCTCCTC	ATCCTCCTTCATCATCTTCATACTC	185
*McYABBY6*	GACTCCCATCTGCTTACAATC	TGTACCTTGCCCAGTTCTTT	120
*McYABBY7*	CCCTACTACCTTCTCGCAACT	ATCGGACGGTGACTGTTT	143
*McYABBY* *8*	TCTTCGCAACAGGGTCATAAA	GTCACGCTCAGCAGAGTCAAT	111

**Table 2:** Basic information of the *McYABBY* genes in bitter gourd.

Gene Name	^a^ Gene ID	^a^ Chromosome Location (bp)	CDS Length (bp)	Protein Length (aa)	^b^ Subcellular Localization
*McYABBY1*	Y52_Chr0100175	Chr01:1,383,521–1,386,579	582	193	Nucleus
*McYABBY2*	Y52_Chr0103109	Chr01:35,327,476–35,328,692	552	183	Nucleus
*McYABBY3*	Y52_Chr0201055	Chr02:9,685,639–9,687,408	699	232	Nucleus
*McYABBY4*	Y52_Chr0202173	Chr02:28,840,451–28,843,209	564	187	Nucleus
*McYABBY5*	Y52_Chr0300164	Chr03:997,373–999,191	606	201	Nucleus
*McYABBY6*	Y52_Chr0300352	Chr03:2,155,421–2,156,833	522	173	Nucleus
*McYABBY7*	Y52_Chr0900327	Chr09:3,188,612–3,194,304	675	224	Nucleus
*McYABBY8*	Y52_Chr1001658	Chr10:20,539,749–20,544,468	510	169	Nucleus

^a^ Information from Bittergourd DB database (http://bittergourddb.omicsplant.cn/); ^b^ subcellular localization of the proteins predicted by the program Cell-PLoc v 2.0.

## Data Availability

All data related to this study are open access, and the databases, websites, and software information used have been detailed in the article and are available for interested researchers.

## Supplementary Materials

The following supporting information can be downloaded at: https://www.mdpi.com/article/10.3390/biology15211938/s1, Table S1. RNA-seq sample information of nine bitter gourd cultivars (from Bittergourd DB).
