# Supplementary material for: Genome-Wide Identification and Expression Analysis of the YABBY Gene Family in Bitter Gourd
Source: Biology (Basel). 2026 Oct 25;15(21):1938. doi: 10.3390/biology15211938 (PMC13762307; doi:10.3390/biology15211938)
Supplement: Supplementary file 1 [file biology-15-01938-s001.zip › McYABBY proteins sequences/McYABBY proteins sequences.pdf]

Momordica charantia

>McYABBY1
MSSCINSQQEQLCYIPCNFCNIVLAVSVPCSNLFDIVTVRCGHCSNLWSVNMAAAFQSLSW
PNSQGSGHTHSHGGSAADYKVELGSSSKCNNKMKMRATIKNPTIDPDQRVINRPPEKRQR
VPSAYNQFIKEEIQRIKAK
NPDITHREAFSTAAKNWAHFPHIHFGLMLETNNLQTKNDGSEKHLMSVPGLHY

>McYABBY2
MSTVEDLLDSSEEIRYVQCGLCSTILLVNVPYSNLSMVVSVRCGNCTGLLSVNMAMPSFIP
FDLLTSLSHNLPKQLAFGQDFDLNNVTSDLIASNHNIIQDHEDDPDLIINIPPTPVVNKPPEK
KQRAPSAYNQFIKEEI
RRLKAENPAMAHKEAFKTAAKNWAHLPPAHGEEGDQMRKKIVV

>McYABBY3
MSSSSSSPTPPPCHPLPPPPPPPLDHHLPAAEQLCYVHCNICDTVLAVSVPCSSLFKTVTVRC
GHCTNLLPVNMRGLLLPSTNQFHQLGHSFFSPSSHNILENIPTPNSNFLINQFAATNNFGMP
SRGVTDELPRPPVINRPPEKRQRVPSAYNRFIKDEIQRIKSVNPDISHREAFSAAAKNWAHF
PHIHFGLMPDQTVKKTNIRQQEGDQNVLMKDNGFYASANVGVSHF

>McYABBY4
MASCIDIAPEQLCYIPCNFCNIVLAVSVPCSSLFDIVTVRCGHCTNLWSVNMAAAFQSLSW
QDVQVPNYNLYSCSGPDFQGDFGSSSKCNNNKMEIRAPTTIPAEERVVNRPPEKRQRVPSA
YNQFIKEEIQRIKANNPEISHREAFSTAAKNWAHFPRIHFGLMLETNNNQVKLDDGLSRAA
LLNK

>McYABBY5
MNHLFDLPEQICYVQCGICTTILLVSVPCSSLSMAVTVTCGHCSSLLSVNMMKATLVPLHF
LSSIARNVPKESGGEMNSGKFLDSFKASNLKFSEYEDDEGGLIPVTPPVVNKPPAKRQRAP
SAYNCFIKNEIRRLKAQNPQMSHKEAFRTAAKNWANFPPIQQKEDAESCNQIEENGVWDT
QMPEVHNDGKCSLRHSVLT

>McYABBY6
MSSEDSKTSMDLVQPSEHLCYVRCNFCNTVLAVGIPCRRLLDTVTVKCGHCSNLSFLSTRP
PLQGQCIDHPLGFQTQVGFSNDIRKGASTSSTSTSSSMANESPSPSFVVKPPEKKHRLPSAY
NRFMKEEIQRIKAANPEIPHREAFSAAAKNWARYIPNSAAGSVSGTHNNE

>McYABBY7
MSSSSLPTTFSQLSPSLPSPPLDQLCYVHCNICDTVLGVSVPPASLFKTVTVRCGHCTNLLP
VNMPPLLPSTDQFHGFARSPAFFPLNTHNFLEEISNPNPNPNLVMNQTDGNDLTMATRVAN
DIPRQPPTINRPPEKRQRVPSAYNRFIKDEIQRIKTVNPDISHREAFSAAAKNWAHFPHINFG
VPPHRTAKKTNMLHQEGDDVTLKDDRFLGSTNMGVSPC

>McYABBY8
MSLDMMASERVCYVHCNFCNTILAVSVPCNSMFTLVTVRCGHCSNLLSVNMGASLQVVP
PQDSSQQGHKQQQVSSTGDSSKERGSSSSKCSKIDSAERDQHRIPPIRPPEKRQRVPSAYNR
FIKEEIQRIKAKNPDISHREAFSTAAKNWAHFPHIHFGLKLDGNKQSN

Fragaria vesca

>FvYABBY1

MSSCIDAVPEQLCYIPCNFCNIVLAVSVPCSSLLDIVTVRCGHCTNLWSVNMAAAFQSLSW
QDVQAQHNHNAQNYRIDSGSSSKGNKKIATRDSIADHRATEERVNRPPEKRQRVPSAYNQ
FIKEEIQRIKANNPDISHREAFSTAAKNWAHFPHIHFGLMLETNNQAKLDNGSEKHLMSRA
AVLK

>FvYABBY2

MSMELMASSERVCYVHCNHCNTILAVSVPAFNGPLTAATTTVVTVRCGHCANLLSVNIGA
ASLQYHSITPSATAAAAAASPHPHPHTQHYHSHPNIFTHQKQHQVSSEDSSSNKDYGSSSS
SSTSSKCNKFAAAPAFDQSSTTVDQPRISPIRPPEKRQRVPSAYNRFIKEEIQRIKASNPDISH
REAFSTAAKNWAHFPHIHFGLKLESNKQAKLDNQTFAGEGTQKSTNGLC

>FvYABBY3

MNLEDKVPMDLVQPSEHLCYVRCNFCNTVLAVGLPFKRMMDTVTVKCGHCSNLSFLTTR
PNNPIQIQPAPHQCQINDYPTTLTLHQSYSTDSRRGGHSSSSSSPTSLESPSSPKAPFVCKPPE
KKHRVPSAYNRFMKEEIQRIKAANPEIPHREAFSAAAKNWARYPSSSGEAAATASGSSKKL
KILQD

>FvYABBY4

MSTLNHLFDLPAEQICYVQCGFCATLLLVSVPCSSLSRLVTVRCGHCSCLLSVNMIKASFLP
FHHLLASLGQDDVDQGSQEKDREICEEEMEKVKYPGLGDMRSQSMVVSNNYENEEDYT
NTFSLNHIVNKPPEKRQRAPSAYNRFIKEEIQRLKSENPSMTHKEAFSTAAKNWAQFPPVQ
YKIDGPDHGESSSQLIREDQNVTWNSDVTDQVQEDGRDFRERKAPRHSIWARTPFE

>FvYABBY5

MSSSNSASSTLSLDHLAPSEQLCYVHCNICDTVLAVSVPCTSLFKTVTVRCGHCTNLLPVN
MRGLLLPSPNQFHHLGHSFFSQPNSHNLLDQEIPLMNPTPNFLMNNQTSSLNDFAVRPRGG
VADELPRPPVINRPPEKRQRVPSAYNRFIKDEIQRIKSVNPDISHREAFSAAAKNWAHFPHIH
FGLMPDQNTSTVKKTNVRQQESAEDVLMKDHNGFFAAANNNVRVSPY

>FvYABBY6

MSAAASSNTVFSPPDHLSPSDQLCYVHCNFCDTVLAVSVPCSSLFKTVTVRCGHCSNLLS
VNMRGLLLPAAANLGHSFFTPQNLLEEIRNAPSNTFTNQPNMMMNESFMPMIRGVEEIPK
PPPVVNRPPEKRQRVPSAYNRFIKEEIQRIKAGNPDISHREAFSAAAKNWAHFPHIHFGLMP
DQPVKKANMRQDGEDMLIKDNGFFNAAANLGVSPY

Solamum lycopersicum
>SlYABBY1
MSSSSPNSSCLNLLESADHKINTMDPSEHLCYVRCSFCNTILAVGIPCKKLLDTVTVKCGH
CGNLSFLSSRPPIQPQFFDHQPILQHQDFFNNFKKGQSSSSSEPSSPKAPFVVKPPEKKHRLP
SAYNRFMKDEIQRIKAAHPQIPHREAFSAAAKNVHSKYSKWDLG
>SlYABBY2
MSSSSAAFAPDHHLHHHHLSPPSGEQLCYVQCNFCDTVLAVSVPCSSFFKNVTVRCGHCT
NLLSVNIILPTAANQLHLPHSFFSPHNLLDEIRNNPPSLLMNQQINPNESLMPVRGGVDEIP
KPPVANRPPEKRQRVPSAYNRFIKDEIQRIKAGNPDISHREAFSAAAKNWAHFPHIHFGLMP
DQPVKKPNACQQEGEDVLMKEGFLAPANIGVSPY
>SlYABBY3
MSTLNNHLFELQDTICYVQCGYCTTILLVSVPCSSLCNKVVTVRCGHCTTLLSLNLIKPSL
HLFASFDQTHQPPEVDKDETDDANKKNSNSDEEDQLENNVLPLNQVVNKPPEKRQRAPS
AYNCFIKEEIKRLKTLYPNMTHKQAFSTAAKNWAHFPPSQHRGGCSLGERKMAKVSAAR
NSMVPRDSNGLIP
>SlYABBY4
MDYVQSSEHLCYVRCNFCNTVLAVGIPYKRLMDTVTVKCGHCSNLSFLTTRPPIQGQCFD
HQPNIQGYCSELKNKGQASSSTSSTSSEPLSPKAPFVVKPPEKKHRLPSAYNRFMKEEIQRI
KSENPEIPHREAFSAAAKNWARYLPNPPNSGNTNNV
>SlYABBY5
MSLDMTYSSSSSERVCYVHCNFCSTILAVSVPCNSMMTIVTVRCGHCANLLSVNIAPSLQS
LPIQDLQRQNESSIEDGMSRGYGSSSSSTNSFHRFSPIPTDHDQPRSPPIRVPEKRQRVPSAY
NRFIKEEIQRIKASNPDISHREAFSTAAKNWAHFPHIHFGLKLDGNKQTNKLDHAVAGEGP
QKTIGLY
>SlYABBY6
MASCIDVASDQLCYIPCNFCNIVLAVSVPCSSLFDIVTVRCGHCTNLWSVNMAAAFQSSSS
ASWQNHQVPNYTAPEYRMDFGSSTKCNMNRMSMRTPITNNVHEERIVNRPPEKRQRVPS
AYNQFIKEEIQRIKANNPDISHREAFSTAAKNWAHFPHIHFGLMLESNNQAKLACGSRKL
>SlYABBY7
MSSSNSLSLDHLPPPPPSEQLCYVHCNVCDTVLAVSVPCTSLFKTVTVRCGHCTNLLPGW
LLPSTNHHHHHFGHTYFSPSHNLLDEISNATPNFLMNQSNSAHEFVQLPARPGFDDLPRPP
PVVNRPPEKRQRVPSAYNRFIKEEIQRIKAGNPDISHREAFSAAAKNWAHFPHIQFGLMPD
QTVKRTNVRQQDGEDVLTKDGLFNTSANVSVSPY
>SlYABBY8
MSFDMTFSSSPSSERVCYLQCNFCNTILAVSVPCSNMLTLVTVRCGHCANMLSVNIGSLIQ
ALPLQDVQKLQRQQYTNVENNSSNYKAYGSSSSSSSKFNRYSSIVSPQIEPKIPSIRPPEKRQ
RVPSAYNRFIKEEIQRIKASNPDISHREAFSTAAKNWAHFPHIHFGLKLEGNK
>SlYABBY9 Solyc12g009580.1.1
MSSSYIDSTNFEKLCYIPCNFCNIVLVVSVPCSNLLDIVTVRCGHCTNLWSVNMAAAFHTN
SWQNHLHHQVGNYTNSPHDQYKVDFASSSITNNSTLEERNVNRPPEKRQRGPSAYNQFIK
EEIQRIKANNPDITHREAFSTAAKNWAHFPHIQFGLMLETDNQAKLGASENKEKLIMHRA
ALPKIKTFTF

Arabidopsis thaliana
>CRC
MNLEEKPTMTASRASPQAEHLYYVRCSICNTILAVGIPLKRMLDTVTVKCGHCGNLSFLTT
TPPLQGHVSLTLQMQSFGGSDYKKGSSSSSSSSTSSDQPPSPSPPFVVKPPEKKQRLPSAYN
RFMRDEIQRIKSANPEIPHREAFSAAAKNWAKYIPNSPTSITSGGHNMIHGLGFGEKK
>FIL   AtYABBY1
MSMSSMSSPSSAVCSPDHFSPSDHLCYVQCNFCQTILAVNVPYTSLFKTVTVRCGCCTNLL
SVNMRSYVLPASNQLQLQLGPHSYFNPQDILEELRDAPSNMNMMMMNQHPTMNDIPSF
MDLHQQHEIPKAPPVNRPPEKRQRVPSAYNRFIKEEIQRIKAGNPDISHREAFSAAAKNWA
HFPHIHFGLVPDNQPVKKTNMPQQEGEDNMVMKEGFYAPAAANVGVTPY
>AtYABBY2
MSVDFSSERVCYVHCSFCTTILAVSVPYASLFTLVTVRCGHCTNLLSLNIGVSLHQTSAPPI
HQDLQPHRQHTTSLVTRKDCASSSRSTNNLSENIDREAPRMPPIRPPEKRQRVPSAYNRFIK
EEIQRIKACNPEISHREAFSTAAKNWAHFPHIHFGLKLDGNKKGKQLDQSVAGQKSNGYY
>AtYABBY3
MSSMSMSSSSAPAFPPDHFSSTDQLCYVHCSFCDTVLAVSVPPSSLFKTVTVRCGHCSNLL
SVTVSMRALLLPSVSNLGHSFLPPPPPPPPNLLEEMRSGGQNINMNMMMSHHASAHHPN
EHLVMATRNGRSVDHLQEMPRPPPANRPPEKRQRVPSAYNRFIKEEIQRIKAGNPDISHRE
AFSAAAKNWAHFPHIHFGLMADHPPTKKANVRQQEGEDGMMGREGFYGSAANVGVAH
N
>INO   AtYABBY4
MTKLPNMTTTLNHLFDLPGQICHVQCGFCTTILLVSVPFTSLSMVVTVRCGHCTSLLSVNL
MKASFIPLHLLASLSHLDETGKEEVAATDGVEEEAWKVNQEKENSPTTLVSSSDNEDEDV
SRVYQVVNKPPEKRQRAPSAYNCFIKEEIRRLKAQNPSMAHKEAFSLAAKNWAHFPPAHN
KRAASDQCFCEEDNNAILPCNVFEDHEESNNGFRERKAQRHSIWGKSPFE
>AtYABBY5
MANSVMATEQLCYIPCNFCNIILAVNVPCSSLFDIVTVRCGHCTNLWSVNMAAALQSLSRP
NFQATNYAVPEYGSSSRSHTKIPSRISTRTITEQRIVNRPPEKRQRVPSAYNQFIKEEIQRIKA
NNPDISHREAFSTAAKNWAHFPHIHFGLMLESNKQAKIA

Oryza sativa

>OsYABBY
MDLVSPSEHLCYVRCTYCNTVLALQVGVPCKRLMDTVTVKCGHCNNLSFLSPRPPMVQP
LSPTDHPLGPFQGPCTDCRRNQPLPLVSPTSNEGSPRAPFVVKPPEKKHRLPSAYNRFMRE
EIQRIKAAKPDIPHREAFSMAAKNWAKCDPRCSSTVSTSNSNPEPRVVAAPIPHQERANEQ
VVESFDIFKQMERSG

>OsYABBY1
MSVQFTSEHVCYVNCNYCNTILVVNVPNNCSYNIVTVRCGHCTMVLSMDLAPFHQARTV
QDHQVQNRGFQ
GNNFGSYDIASRNQRTSTAMYPMPTSQQQVSPIRPPEKRQRVPSAYNRFIKEEIQRIKTSNP
EISHREAFSAAAKNWAHLPRLHFGLSVADGGGGGGSN

>OsYABBY2
MSAQIVPAPEHVCYVHCNFCNTIFAVSVPSNSMLNIVTVRCGHCTSLLSVNLRGLVQALPA
EDHLQDNLKMHNMSFRENYSEYGSSSRYGRVPMMFSKNDTEHMLHVRPPEKRQRVPSA
YNRFIKEEIRRIKANNPDISHREAFSTAAKNWAHFPNIHFGLGSHESSKKLDEAIGAPSPQK
VQRLY

>OsYABBY3
MSSSSSSSASSAAAAAFRPAVVQREQQVVEEKFPAAAAAMREMVLPPVAAAAADSEQEQ
LCYVHCHYCDTVLVVSDLRSNHAHFLSICVHLALDSRAFLVHPWRRVLQVSVPSSSLFET
VTVRCGHCSSLLTVNMRGLLLPTTAAAAPPPPPPPPPPPPPPPAAHFPHSLNLAPANPPHHHS
LLDEISTANSPTQLLLEQHGLGGLMASAASCRNNNSPAAAAAPPPPTSQGKAAAKEPSPRT
NTAVINRPPEKRQRVPSAYNRFIKDEIQRIKAGNPDISHREAFSAAAKNWAHFPHIHFGLMP
DHQGLKKTSLLPQDHQRKDGLLKEGLYAAAAAAAAAAANMGVAPY

>OsYABBY4
MSSSSSSSAVFPLDHLAAPSPTEQLCYVHCNCCDTILAVGVPCSSLFKTVTVRCGHCANLL
SVNLRGLLLPAPAPAPANQLHFGPSLLSPTSPHGLLDEVAFQTPSLLMEQAASASLSSITGRS
SSSCASNAPAMQMPPAKPVQQEPELPKNAPASANRPPEKRQRVPSAYNRFIKDEIQRIKAG
NPDISHREAFSAAAKNWAHFPHIHFGLMPDQGFKKTFKPQDGSEDILLKDSLYAAAAAAA
AAAANMGVTPF

>OsYABBY5
MMSSAPETFSLDHLSQHQQQQPPPLAEQEQLCYVHCNFCDTILAVGVPCSSLFKTVTVRC
GHCANLLSVNLRGLLLPAAASTANQLPFGQALLSPTSPHGLLDEVPSFQAPASLMTEQASP
NVSSITSSNSSCANNAPATSMASAANKATQREPQQPKNAPSANRTSEKRQRVPSAYNRFIK
DEIQRIKASNPDITHREAFSAAAKNWAHFPHIHFGLMPDQGLKKTGIQSQDGAGECMLFK
DGLYAAAAAAAAATAASSMGVTPF

>OsYABB6
MSAQIAPAEQVCYVHCNFCNTILAVSVPGNSMLNIVTVRCGHCTNLLSVNLRGLMHSAPA
LQDHHHHHLQESGLSGCFRDQSGYPEFGFSAASSSSKLRLPPAAAAMVSYSQQNQQLEQ
ALHARPPEKRQRVPSAYNRFIKEEIRRIKANNPDISHREAFSTAAKNWAHYPNIHFGLSPGH
EGGKKLVDVDPIPTAPSSKKIQGFYS

>OsYABB7
MSSAARHHCSGLRERLGCVQCSFCATVLLVSVPCSSVLRVVAVQCGHCSGILSAVNLPPSP
VSASIELTPQELDAGPPPGEYSDESSGDDREGRDAEDDAPAPAAAAVANKPPGRKQRTPSA
YNCFVKEEIKRIKSMEPNITHKQAFSTAAKNWAHLPRIQQKRGRDSC

Punica granatum

>PgCRC
MEDKGPLDSVPPSSDHLCYVRCNFCNTVLAVGIPCKRTLETVTVKCGHCSNLSFLSTRPPI
QGQCLDHHLSLPGYGTEVRKGQSSSSSSSTSSEPQSPKAAPFVAKPPEK
KHRLPSAYNRFMK
>PgINO
MGSINPLFEFSEKICYVQCGFCTTILLVSVPSSSQSMVVTVRCGHCSSLLSVNMMKASFIPL
HLLASLSHDEVEAKAEHPTDQAGTRKNPERRSPSVVTSNSDNEDEDLISVNRTVNKPPEK
RQRAPSAYNRFIKEEIRRLKAQNPNMAHKEAFSQAAKNVS
>PgFILa
MMSSSSSSSSSSLSPDHLSPTDQLRYVHCNFCDTVLAVSVPCSSLFKTVTVRCGHCANLLS
VNMRGLLLPSPAATAATQLQQLGHTFFAPHNLLEEIRNVPPTVLMNHHFPNPAESVLQLHG
GSEPIPRPALVNRPPEKRQRVPSAYNRFIKEEIQRIKAQNPDISHREAFSAAAKNWAHFPHIH
FGLMPEQSVKKPTMCQQEGEDMLRKDGLFAPANLGVSPY
>PgYAB2
MSLEMASERVCYVQCNFCNTILAVSVPCSSMYTVVTVRCGHCANLLTVNMAGASLQPLP
LQELQKQQHLIGPEELMKGSGPSSSKCNTNNNTHNKCTSSLESAPHHQQQQDQPPPPRIPP
IRPPEKRQRVPSAYNRFIKEEIQRIKASNPDISHREAFSTAAKNWAHFPHIHFGLKLEGNMN
KQSKLDHHSSINGGDHHLHPKTSHGFY
>PgYAB5
MSSCVDIAATPDQLCYIPCNFCHIVLAVSVPCSSLLDVVTIRCGHCANLWSVNMAAAYHSS
SSSSSSSSWPDLHHHQIPKQDYRIDSASSSKSKNKVAAVRAPPPKLNEERVVNRPPEKRQR
VPSAYNQFIKEEIQRIKANNPDISHREAFSTAAKNWAHFPHIHFGLMLQTNNQPQLESSAE
KHVMMPRAALHK
>PgFILb
MESNGHGLVTFEAVVRDTVSVPCSSLFKTVTVRCGHCTNLLSVNMRALLLPAPASHQLHL
GHTYFTPQNLLEEIRNAPTNMMINPPIPNESMMQLRGGIDDIPKPPVVNRPPEKRQRVPSA
YNRFIKDEIQRIKAGNPDISHREAFSAAAKNWAHFPHIHFGLMSDQPVKKPNLRQQEGED
MLNMKDGFFPPTANVGVSPY

Citrullus lanatus
>CLaYABBY1
MSSSSSSSSPTSTTTATTTTTLSQQQHLQQQLDHHLPSPEQLCYVHCNICDTVLAVSVPCCS
LFKTVTVRCGHCTNLLPVNMRGLLLPSPNQFHQLGHSFFSPNSHNILENMATPNSNYLINQ
FGATTNNFGMPSRGVTDELPRPPAINRPPEKRQRVPSAYNRFIKDEIQRIKSVNPDISHREAF
SAAAKNWAHFPHIHFGLMPDQTVKKTNIRQQEGDTQNVLMKDNGFYASANVGVSHF
>CLaYABBY2
MSLDMMASERVCYVHCNFCNTILAVSVPCNSMFTLVTVRCGHCSNLLSVNMGASLQVVP
PQDSQQGHKQQQVNAGDSSKDRASSSSSSSSSKCTKIGSLDSAERDQHRIPPIRPPEKRQRV
PSAYNRFIKEEIQRIKAKNPDISHREAFSTAAKNWAHFPHIHFGLKLDGNKQTK
>CLaYABBY3
MSNSEDKSSMDLVQPSDHLCYVKCNFCNTLLAVGIPCRRLLDTVTVKCGHCSNLSFLSTR
PPLQGQCIDHPLSFQSQVGFSNDIRKGASTSSTSTSSSMPNESPSPNFVVKPPEKKHRLPSA
YNRFMKEEIQRIKAANPEIPHREAFSAAAKNWARYIPNSAAGSVSGTHHNNE
>CLaYABBY4
MSNSEDKSSMDLVQPSDHLCYVKCNFCNTLLAVGIPCRRLLDTVTVKCGHCSNLSFLSTR
PPLQGQCIDHPLSFQSQVGFSNDIRKGASTSSTSTSSSMPNESPSPNFVVKPPEKKHRLPSA
YNRFMKEEIQRIKAANPEIPHREAFSAAAKNWARYIPNSAAGSVSGTHHNNE
>CLaYABBY5
MNHLFELPEKICYVQCGICTTILLVSVPCGSLSMAVTVTCGHCSSLLSVNMMKATLVPLHF
LSSLSRNVPKEACREMNSGKFSDSFKRPNLKFSEYEVEEGLIPATPPFVNKPPEKRQRAPSA
YNCFIKDEIRRLKAHNPEMTHKEAFRTAAKNWANFPPIQEKEDTEN
>CLaYABBY6
MAASCIDIAPEQLCYIPCNFCNIVLAVSVPCSSLFDIVTVRCGHCTNLWSVNMAAAFQSLS
WQDLQVPNYNLYGCSGGDFQGDFGSSSKCNNSNKMEIRAPTTIAAEERVVNRPPEKRQR
VPSAYNQFIKEEIQRIKASNPEISHREAFSTAAKNWAHFPRIHFGLMLETNNQVKLDDGSER
LMTRAALLNK
>CLaYABBY7
MSTVEQLLADSSEQIRYVQCGLCSTILLVNVPYSNLSMVVSVRCGNCSGLLSVNMAKPSFI
PFDLLTSLSHNLPKEPFAQEFNVGQKHYCLDSLSNTSDLITSYHNIIHNHEDDDDVIIIPPTP
VVNKPPEKKQRAPSAYNQFIKEEIRRLKAENPAMAHKEAFKTAAKNWAHLPPVNAAAGV
DHMTMRKKNVM
>CLaYABBY8
MSSSSIPNTFSQVPPPLLPPPSSEQLCYVHCNICDTVLAVSVPCSSLFKRVTVRCGYCANLLP
VNMCGGMLLPSSNQFHGFAHSTPNFLSPNTHNFLEEISNSNPNPNPNFLMNQAEGIDLTMA
TRVANDLPRQPPSINRPPEKRQRVPSAYNRFIKEEIQRIKAANPDISHREAFSAAAKNVSRY
II
>CLaYABBY9
MSSCINSAQEQLCYIPCNFCNIVLAVSVPCNNLFDIVTVRCGHCSNLWSVNMAAAFQSLS
WQNSQASNHSHSGSGADHYRVELGSSSKCNNKMKMRAPIKNSTTDHDQRIINRPPEKRQ
RVPSAYNQFIKEEIQRIKATNPDITHREAFSTAAKNWAHFPHIHFGLMLDTNNLQTKNDGS
EKHLMSVPGLHY
