# Supplementary material for: Genome-Wide Identification and Expression Analysis of the YABBY Gene Family in Bitter Gourd
Source: Biology (Basel). 2026 Oct 25;15(21):1938. doi: 10.3390/biology15211938 (PMC13762307; doi:10.3390/biology15211938)
Supplement: Supplementary file 1 [file biology-15-01938-s001.zip › Table S1.pdf]

Table S1. RNA-seq sample information of nine bitter gourd cultivars (from Bittergourd DB).

| Sample | Organ | Cultivar (code) | Developmental stage | Replicates | BioProject | GSA accession |
|---|---|---|---|---|---|---|
| F | Female flower | 1, 2, 10, 13, 62, 66, 92, 110, 124 | Flowering stage | 3 | PRJCA036725 | CRA023647 |
| LS | Leaf and shoot | 1, 2, 10, 13, 62, 66, 92, 110, 124 | Vegetative stage | 3 | PRJCA036725 | CRA023647 |
| SF | Small fruit | 1, 2, 10, 13, 62, 66, 92, 110, 124 | Early fruit development (descriptive category, not a fixed DAP) | 3 | PRJCA036725 | CRA023647 |
| BF | Big fruit | 1, 2, 10, 13, 62, 66, 92, 110, 124 | Late fruit development (descriptive category, not a fixed DAP) | 3 | PRJCA036725 | CRA023647 |

Cultivar codes correspond to the Bittergourd DB sample IDs (e.g., F110-1/2/3 denote three biological replicates of female flower from cultivar 110, the reference inbred line Y52). The developmental stages "Small fruit" (SF) and "Big fruit" (BF) are descriptive categories in Bittergourd DB and do not correspond to strictly defined DAP (days after pollination) stages.
